# Supplementary material for: Effectiveness of BBIBP-CorV vaccine against severe outcomes of COVID-19 in Abu Dhabi, United Arab Emirates
Source: Nat Commun. 2022 Jun 9;13:3215. doi: 10.1038/s41467-022-30835-1 (PMC9184465; doi:10.1038/s41467-022-30835-1)
Supplement: Supplementary file 1 — Supplementary Information [file 41467_2022_30835_MOESM1_ESM.pdf]

Supplementary material

Effectiveness of inactivated SARS-CoV-2 vaccine against COVID-19 related hospital/critical care admissions and mortality in the Emirate of Abu Dhabi, United Arab Emirates

Contents

1 - PRIMARY ANALYSIS 2

Supplementary Table 1 COVID-19 Severe outcomes according to risk factors and vaccine status: Primary analysis 3

Supplementary Table 2. Results of fitting the multiple Cox Proportional Hazard model to the three outcomes: Primary analysis. 4

Supplementary Table 3. Interactions between Vaccine status and risk factors: Primary analysis........ 5

Supplementary Table 4. Effectiveness using time varying coefficient model with a step function: Primary analysis 8

Supplementary Figure 1. Schoenfeld residuals for testing proportionality of the Cox proportional hazard model: Primary analysis........ 9

2- SENSITIVITY ANALYSIS 10

Supplementary Table 5. Demographic and Clinical Characteristics at baseline of Vaccinated and Unvaccinated individuals: Sensitivity analysis11

Supplementary Table 6. COVID-19 Severe outcomes according to risk factors and vaccine status: Sensitivity analysis 13

Supplementary Table 7. Results of fitting the multiple Cox Proportional Hazard model to the three outcomes: Sensitivity analysis 14

Supplementary Table 8. Interactions between Vaccine status and risk factors: Sensitivity analysis........ 15

Supplementary Table 9. Effectiveness of the Vaccine in Preventing Severe COVID-19 Outcomes stratified according to different risk factors: Sensitivity analysis 18

Supplementary Table 10. Effectiveness using time varying coefficient model with a step function: Sensitivity analysis 19

Supplementary Figure 2. Schoenfeld residuals for testing proportionality of the Cox proportional hazard model: Sensitivity analysis........ 20

3- Ethnicity of the study participants........21

Supplementary Table 11: Ethnicity of the study participants 21

1 - PRIMARY ANALYSIS

Supplementary Table 1 COVID-19 Severe outcomes according to risk factors and vaccine status: Primary analysis

Risk factors	Unvaccinated, Hospitalized	Vaccinated, Hospitalized	Unvaccinated, Critical	Vaccinated, Critical	Unvaccinated, Died	Vaccinated, Died
**Age group n (%)**						
Less than 40 years	834 (0.1)	128 (<0.1)	83 (<0.1)	12 (<0.1)	0 (0)	0 (0)
40-60 years	924 (0.3)	190 (<0.1)	198 (<0.1)	33 (<0.1)	21 (<0.1)	2 (<0.1)
More than 60 years	1,078 (1.8)	279 (0.7)	283 (0.5)	32 (<0.1)	65 (0.1)	10 (<0.1)
**Sex n (%)**						
Female	1,716 (0.4)	309 (<0.1)	228 (<0.1)	41 (<0.1)	34 (<0.1)	7 (<0.1)
Male	1,120 (0.2)	288 (<0.1)	336 (<0.1)	36 (<0.1)	52 (<0.1)	5 (<0.1)
**Comorbidities n (%)**						
Without any comorbidity	810 (<0.1)	239 (<0.1)	129 (<0.1)	28 (<0.1)	13 (<0.1)	2 (<0.1)
With one or more comorbidities	2,026 (3.6)	358 (0.5)	435 (0.8)	49 (<0.1)	73 (0.1)	10 (<0.1)
**Ethnicity n (%)**						
Arab	2,038 (0.5)	448 (0.1)	375 (<0.1)	55 (<0.1)	70 (<0.1)	9 (<0.1)
Asian	664 (0.1)	105 (<0.1)	1473 (<0.1)	20 (<0.1)	14 (<0.1)	3 (<0.1)
Other	134 (0.1)	44 (<0.1)	42 (<0.1)	2 (<0.1)	2 (<0.1)	0 (0)
**Month of observation n (%)**						
Oct-Dec 2020	786 (1.2)	18 (<0.1)	203 (0.3)	2 (<0.1)	26 (<0.1)	0 (0)
Jan-Apr 2021	1,922 (0.2)	500 (<0.1)	341 (<0.1)	67 (<0.1)	51 (<0.1)	9 (<0.1)
May-Jul 2021	128 (<0.1)	79 (<0.1)	20 (<0.1)	8 (<0.1)	9 (<0.1)	3 (<0.1)

Supplementary Table 2. Results of fitting the multiple Cox Proportional Hazard model to the three outcomes: Primary analysis.

Risk factor	n (%)	Hospitalization			Critical care admission			Death		
		Event N	HR (95% CI)	p-value	Event N	HR (95% CI)	p-value	Event N	HR (95% CI)	p-value
**Vaccination status**				<0.001			<0.001			<0.001
Unvaccinated	1,099,886 (50)	2836	—		564	—		86	—	
Vaccinated	1,099,886 (50)	597	0.20 (0.19 to 0.22)		77	0.14 (0.11 to 0.18)		12	0.16 (0.09 to 0.29)	
**Age group**				<0.001			<0.001			<0.001
≤ 60 years	2,096,204 (95)	2076	—		326	—		23	—	
> 60 years	103,568 (4.7)	1357	4.23 (3.93 to 4.55)		315	5.82 (4.94 to 6.87)		75	18.1 (11.1 to 29.8)	
**Sex**				<0.001			<0.001			0.025
Female	1,400,773 (64)	1408	—		372	—		57	—	
Male	798,999 (36)	2025	1.50 (1.39 to 1.61)		269	0.73 (0.62 to 0.86)		41	0.63 (0.42 to 0.95)	
**Comorbidity**				<0.001			<0.001			<0.001
Without any comorbidity	2,076,268 (94)	1049	—		157	—		15	—	
With one or more comorbidities	123,504 (5.6)	2384	21.9 (20.1 to 23.8)		484	36.4 (29.6 to 44.8)		83	37.2 (20.0 to 69.1)	
**Ethnicity**				<0.001			0.008			0.29
Arab	760,211 (35)	2486	—		430	—		79	—	
Non-Arab	1,439,561 (65)	947	0.80 (0.73 to 0.87)		211	1.29 (1.07 to 1.54)		19	0.75 (0.44 to 1.30)	
**Month of observation**				<0.001			<0.001			<0.001
Oct-Dec 2020	131,850 (6.0)	804	—		205	—		26	—	
Jan-Apr 2021	1,774,370 (81)	2422	0.32 (0.29 to 0.35)		408	0.23 (0.19 to 0.27)		60	0.29 (0.18 to 0.46)	
May-Jul 2021	293,552 (13)	207	0.19 (0.16 to 0.22)		28	0.12 (0.08 to 0.18)		12	0.49 (0.25 to 0.98)	

HR= Hazard Ratio, CI = Confidence Interval. P-value for testing the association between vaccine status and outcomes adjusting for risk factors. P-values were calculated using two-sided tests with no adjustment for multiple comparisons, and were computed using the Wald test on the coefficients of the Cox proportional hazard model. P-values < 0.05 are considered statistically significant.

Supplementary Table 3. Interactions between Vaccine status and risk factors: Primary analysis

Outcome: Hospitalization

	**Coefficient**	**Standard error**	**P-values**
**Comorbidity (Reference: Without comorbidity)**			
Vaccine effect	-1.16	0.0736	<0.0001
Comorbidity effect	3.2	0.0477	<0.0001
Interaction effect	-0.641	0.0934	<0.0001
**Age (Reference: age < 60)**			
Vaccine effect	-1.87	0.061	<0.0001
Age effect	1.32	0.0406	<0.0001
Interaction effect	0.722	0.0908	<0.0001
**Sex (Reference: Male)**			
Vaccine effect	-1.41	0.0663	<0.0001
Sex effect	0.461	0.0398	<0.0001
Interaction effect	-0.324	0.0906	0.0003
**Ethnicity (Reference: Arab)**			
Vaccine effect	-1.58	0.0524	<0.0001
Ethnicity effect	-0.216	0.0464	<0.0001
Interaction effect	-0.0545	0.103	0.59
**Month of observation (Reference: Oct-Dec 2020)**			
Vaccine effect	-3.67	0.239	<0.0001
Jan-Apr 2021 effect	-1.32	0.0432	<0.0001
May-Jul 2021 effect	-2.1	0.096	<0.0001
Interaction Jan-Apr 2021	2.25	0.244	<0.0001
Interaction May-Jul 2021	3.13	0.278	<0.0001

Outcome: Critical care admission

	**Coefficient estimate**	**Standard error**	**P-values**
**Comorbidity (Reference: Without comorbidity)**			
Vaccine effect	-1.44	0.209	<0.0001
Comorbidity effect	3.7	0.113	<0.0001
Interaction effect	-0.734	0.257	0.004
**Age (Reference: age < 60)**			
Vaccine effect	-2.02	0.161	<0.0001
Age effect	1.75	0.089	<0.0001
Interaction effect	0.123	0.246	0.617
**Sex (Reference: Male)**			
Vaccine effect	-2.24	0.176	<0.0001
Sex effect	-0.383	0.0882	<0.0001
Interaction effect	0.591	0.244	0.015
**Ethnicity (Reference: Arab)**			
Vaccine effect	-1.93	0.145	<0.0001
Ethnicity effect	0.264	0.098	0.007
Interaction effect	-0.116	0.268	0.665
**Month of observation (Reference: Oct-Dec 2020)**			
Vaccine effect	-4.46	0.711	<0.0001
Jan-Apr 2021 effect	-1.62	0.0906	<0.0001
May-Jul 2021 effect	-2.42	0.236	<0.0001
Interaction Jan-Apr 2021	2.78	0.723	<0.0001
Interaction May-Jul 2021	3.54	0.825	<0.0001

Outcome: Death

	**Coefficient estimate**	**Standard error**	**P-values**
**Comorbidity (Reference: Without comorbidity)**			
Vaccine effect	-0.934	0.411	0.0229
Comorbidity effect	2.94	0.241	<0.0001
Interaction effect	-2.6	0.781	0.0009
**Age (Reference: age < 60)**			
Vaccine effect	-2.57	0.74	0.0005
Age effect	2.78	0.264	<0.0001
Interaction effect	0.953	0.815	0.242
**Sex (Reference: Male)**			
Vaccine effect	-2.2	0.469	<0.0001
Sex effect	-0.558	0.225	0.0132
Interaction effect	0.727	0.626	0.246
**Ethnicity (Reference: Arab)**			
Vaccine effect	-1.94	0.355	<0.0001
Ethnicity effect	-0.346	0.297	0.244
Interaction effect	0.472	0.722	0.513
**Month of observation (Reference: Oct-Dec 2020)**			
Vaccine effect	-16.3	0.308	<0.0001
Jan-Apr 2021 effect	-1.35	0.207	<0.0001
May-Jul 2021 effect	-0.965	0.308	0.0017
Interaction Jan-Apr 2021	14.6	0.35	<0.0001
Interaction May-Jul 2021	15.4	0.586	<0.0001

P-value for testing the main and interaction effects between each risk factors and vaccine status. P-values were calculated using two-sided tests with no adjustment for multiple comparisons, and were computed using the Wald test on the coefficients of the Cox proportional hazard model. P-values < 0.05 are considered statistically significant.

Supplementary Table 4. Effectiveness using time varying coefficient model with a step function: Primary analysis

Time period	Hospitalization			Critical care admission		
	Effectiveness (%)	95% CI	p-value	Effectiveness (%)	95% CI	p-value
Prior to day 57	82.5	80.4 , 84.3	<0.001	87.4	83 , 90.6	<0.001
After day 57	71	66.3 , 75.1	<0.001	78.6	67.9 , 85.8	<0.001

Cox proportional hazard model with time varying coefficients fitted to the data. HR = Hazard Ratio, CI = Confidence Interval. P-values were calculated using two-sided tests with no adjustment for multiple comparisons, and were computed using the Wald test on the coefficients of the Cox proportional hazard model. P-values < 0.05 are considered statistically significant.

Supplementary Figure 1. Schoenfeld residuals for testing proportionality of the Cox proportional hazard model: Primary analysis

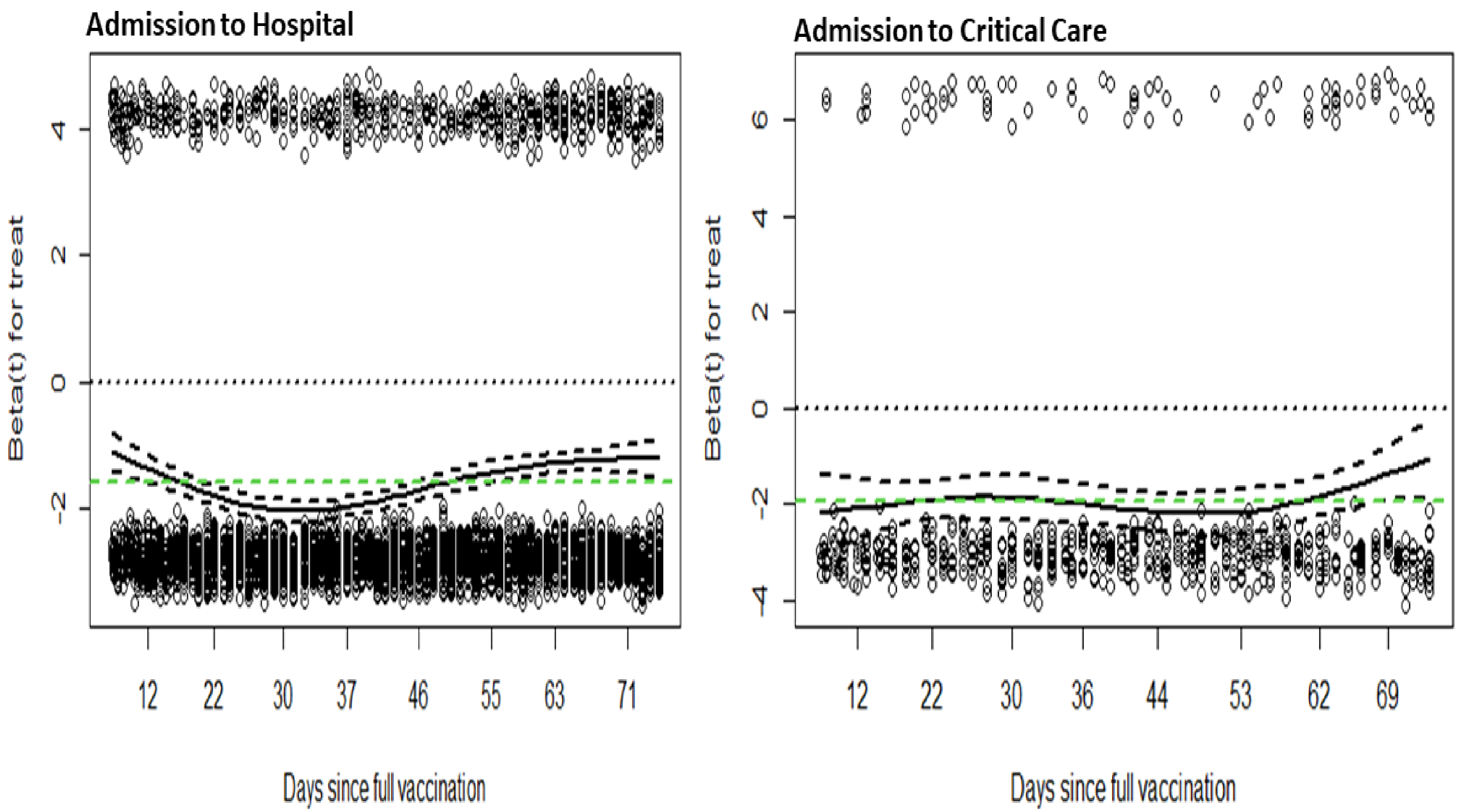

The solid black curve represents the estimated time varying log-hazard ratio with the dashed black curves representing the corresponding 95% confidence limits. The green dashed line represents the estimated log-hazard ratio obtained from the Cox proportional hazard model (under the assumption of proportional hazards).

2- SENSITIVITY ANALYSIS

Supplementary Table 5. Demographic and Clinical Characteristics at baseline of Vaccinated and Unvaccinated individuals: Sensitivity analysis

Characteristic	**Overall,** N = 2,276,996[1]	**Unvaccinated**, N = 1,138,498	**Vaccinated**, N = 1,138,498
**Median age (IQR)**	35 (28 – 43)	34 (28 – 43)	35 (29 – 43)
**Age group n (%)**			
Less than 40 years	1,501,894 (66)	756,547 (66)	745,347 (65)
40-60 years	668,600 (29)	318,951 (28)	349,649 (31)
More than 60 years	106,502 (4.7)	63,000 (5.5)	43,502 (3.8)
**Sex n (%)**			
Female	844,775 (37)	421,339 (37)	423,436 (37)
Male	1,432,221 (63)	717,159 (63)	715,062 (63)
**Ethnicity n (%)**			
Arab	808,970 (36)	400,530 (35)	408,440 (36)
Asian	1,232,786 (54)	617,322 (54)	615,464 (54)
Other	235,240 (10)	120,646 (11)	114,594 (10)
**Comorbidities n (%)**			
Asthma	29,571 (1.3)	13,651 (1.2)	15,920 (1.4)
Chronic Kidney disease	19,270 (0.8)	9,069 (0.8)	10,201 (0.9)
Diabetes	98,081 (4.3)	47,708 (4.2)	50,373 (4.4)
Heart disease	15,155 (0.7)	6,993 (0.6)	8,162 (0.7)
Hypertension	53,156 (2.3)	25,460 (2.2)	27,696 (2.4)
Immunodeficiencies	2,989 (0.1)	1,483 (0.1)	1,506 (0.1)

Neoplasms	21,702 (1.0)	10,821 (1.0)	10,881 (1.0)
Respiratory diseases	3,854 (0.2)	1,841 (0.2)	2,013 (0.2)
History of Transplantation	1,433 (<0.1)	721 (<0.1)	712 (<0.1)
**Comorbidities n (%)**			
No comorbidity	2,134,301 (94)	1,073,252 (94)	1,061,049 (93)
One comorbidity	82,318 (3.6)	35,610 (3.1)	46,708 (4.1)
2 or more comorbidities	60,377 (2.7)	29,636 (2.6)	30,741 (2.7)
**Month of observation n (%)**			
Oct-Dec 2020	141,870 (6.2)	70,935 (6.2)	70,935 (6.2)
Jan-April 2021	1,833,056 (81)	916,528 (81)	916,528 (81)
May-July 2021	302,070 (13)	151,035 (13)	151,035 (13)
**Month of observation n (%)**			
Oct-20	1,334 (<0.1)	667 (<0.1)	667 (<0.1)
Nov-20	69,578 (3.1)	34,789 (3.1)	34,789 (3.1)
Dec-20	70,958 (3.1)	35,479 (3.1)	35,479 (3.1)
Jan-21	257,894 (11)	128,947 (11)	128,947 (11)
Feb-21	827,854 (36)	413,927 (36)	413,927 (36)
Mar-21	405,514 (18)	202,757 (18)	202,757 (18)
Apr-21	119,026 (5.2)	59,513 (5.2)	59,513 (5.2)
May-21	222,768 (9.8)	111,384 (9.8)	111,384 (9.8)
Jun-21	197,862 (8.7)	98,931 (8.7)	98,931 (8.7)
Jul-21	104,208 (4.6)	52,104 (4.6)	52,104 (4.6)

Supplementary Table 6. COVID-19 Severe outcomes according to risk factors and vaccine status: Sensitivity analysis

Characteristic	Unvaccinated, Hospitalized	Vaccinated, Hospitalized	Unvaccinated, Critical	Vaccinated, Critical	Unvaccinated, Died	Vaccinated, Died
**Age group n (%)**						
≤ 60 years	1,758 (0.2)	349 (<0.1)	281 (<0.1)	50 (<0.1)	21 (<0.1)	2 (<0.1)
More than 60 years	1,078 (1.7)	301 (0.7)	283 (0.4)	36 (<0.1)	65 (0.1)	10 (<0.1)
**Age group n (%)**						
Less than 40 years	834 (0.1)	141 (<0.1)	83 (<0.1)	14 (<0.1)	0 (0)	0 (0)
40-60 years	924 (0.3)	208 (<0.1)	198 (<0.1)	36 (<0.1)	21 (<0.1)	2 (<0.1)
More than 60 years	1,078 (1.7)	301 (0.7)	283 (0.4)	36 (<0.1)	65 (0.1)	10 (<0.1)
**Sex n (%)**						
Female	1,716 (0.4)	343 (<0.1)	228 (<0.1)	47 (<0.1)	34 (<0.1)	7 (<0.1)
Male	1,120 (0.2)	307 (<0.1)	336 (<0.1)	39 (<0.1)	52 (<0.1)	5 (<0.1)
**Comorbidities n (%)**						
Without any comorbidity	810 (<0.1)	252 (<0.1)	129 (<0.1)	30 (<0.1)	13 (<0.1)	2 (<0.1)
With 1+ comorbidities	2,026 (3.1)	398 (0.5)	435 (0.7)	56 (<0.1)	73 (0.1)	10 (<0.1)
**Ethnicity n (%)**						
Arab	2,038 (0.5)	493 (0.1)	375 (<0.1)	64 (<0.1)	70 (<0.1)	9 (<0.1)
Asian	664 (0.1)	113 (<0.1)	147 (<0.1)	20 (<0.1)	14 (<0.1)	3 (<0.1)
Other	134 (0.1)	44 (<0.1)	42 (<0.1)	2 (<0.1)	2 (<0.1)	0 (0)
**Ethnicity n (%)**						
Arab	2,038 (0.5)	493 (0.1)	375 (<0.1)	64 (<0.1)	70 (<0.1)	9 (<0.1)
Non-Arab	798 (0.1)	157 (<0.1)	189 (<0.1)	22 (<0.1)	16 (<0.1)	3 (<0.1)
**Month of observation n (%)**						
Oct-Dec 2020	786 (1.1)	19 (<0.1)	203 (0.3)	2 (<0.1)	26 (<0.1)	0 (0)
Jan-Apr 2021	1,922 (0.2)	544 (<0.1)	341 (<0.1)	74 (<0.1)	51 (<0.1)	9 (<0.1)
May-Jul 2021	128 (<0.1)	87 (<0.1)	20 (<0.1)	10 (<0.1)	9 (<0.1)	3 (<0.1)

Supplementary Table 7. Results of fitting the multiple Cox Proportional Hazard model to the three outcomes: Sensitivity analysis

Characteristic	n (%)	Hospitalization			Critical care admission			Death		
		Event N	HR (95% CI)	p-value	Event N	HR (95% CI)	p-value	Event N	HR (95% CI)	p-value
**Vaccination status**				<0.001			<0.001			<0.001
Unvaccinated	1,138,498 (50)	2836	—		564	—		86	—	
Vaccinated	1,138,498 (50)	650	0.23 (0.21 to 0.25)		86	0.16 (0.13 to 0.20)		12	0.16 (0.09 to 0.29)	
**Age group**				<0.001			<0.001			<0.001
≤ 60 years	2,170,494 (95)	2107	—		331	—		23	—	
More than 60 years	106,502 (4.7)	1379	4.65 (4.33 to 5.00)		319	6.32 (5.37 to 7.44)		75	19.6 (12.0 to 32.0)	
**Sex**				<0.001			<0.001			0.017
Female	1,432,221 (63)	1427	—		375	—		57	—	
Male	844,775 (37)	2059	1.44 (1.34 to 1.54)		275	0.71 (0.61 to 0.84)		41	0.61 (0.40 to 0.92)	
**Comorbidity**				<0.001			<0.001			<0.001
Without any comorbidity	2,134,301 (94)	1062	—		159	—		15	—	
With one or more comorbidities	142,695 (6.3)	2424	19.9 (18.3 to 21.7)		491	33.5 (27.3 to 41.2)		83	34.9 (18.8 to 64.9)	
**Ethnicity**				<0.001			0.003			0.38
Arab	808,970 (36)	2531	—		439	—		79	—	
Non-Arab	1,468,026 (64)	955	0.84 (0.77 to 0.91)		211	1.33 (1.11 to 1.60)		19	0.79 (0.46 to 1.35)	
**Month of observation**				<0.001			<0.001			<0.001
Oct-Dec 2020	141,870 (6.2)	805	—		205	—		26	—	
Jan-Apr 2021	1,833,056 (81)	2466	0.32 (0.30 to 0.35)		415	0.23 (0.19 to 0.27)		60	0.29 (0.18 to 0.46)	
May-Jul 2021	302,070 (13)	215	0.20 (0.17 to 0.23)		30	0.13 (0.09 to 0.19)		12	0.50 (0.25 to 1.00)	

HR= Hazard Ratio, CI = Confidence Interval. P-value for testing the association between vaccine status and outcomes adjusting for risk factors. P-values were calculated using two-sided tests with no adjustment for multiple comparisons, and were computed using the Wald test on the coefficients of the Cox proportional hazard model. P-values < 0.05 are considered statistically significant.

Supplementary Table 8. Interactions between Vaccine status and risk factors: Sensitivity analysis

Outcome: Hospitalization

	Coefficient estimate	Standard error	P-values
**Comorbidity (Reference: Without comorbidity)**			
Vaccine effect	-1.11	0.0722	<0.0001
Comorbidity effect	3.11	0.0477	<0.0001
Interaction effect	-0.571	0.0906	<0.0001
**Age (Reference: age < 60)**			
Vaccine effect	-1.75	0.0587	<0.0001
Age effect	1.41	0.0404	<0.0001
Interaction effect	0.666	0.0878	<0.0001
**Sex (Reference: Male)**			
Vaccine effect	-1.34	0.0646	<0.0001
Sex effect	0.412	0.0399	<0.0001
Interaction effect	-0.267	0.0875	0.0023
**Ethnicity (Reference: Arab)**			
Vaccine effect	-1.46	0.0503	<0.0001
Ethnicity effect	-0.157	0.0465	<0.0001
Interaction effect	-0.12	0.101	0.234
**Month of observation (Reference: Oct-Dec 2020)**			
Vaccine effect	-3.6	0.232	<0.0001
Jan-Apr 2021 effect	-1.32	0.0432	<0.0001
May-Jul 2021 effect	-2.07	0.096	<0.0001
Interaction Jan-Apr 2021	2.28	0.237	<0.0001
Interaction May-Jul 2021	3.18	0.271	<0.0001

Outcome: Critical care admission

	**Coefficient estimate**	**Standard error**	**P-values**
**Comorbidity (Reference: Without comorbidity)**			
Vaccine effect	-1.37	0.203	<0.0001
Comorbidity effect	3.61	0.113	<0.0001
Interaction effect	-0.668	0.248	0.007
**Age (Reference: age < 60)**			
Vaccine effect	-1.89	0.154	<0.0001
Age effect	1.83	0.0886	<0.0001
Interaction effect	0.0914	0.235	0.697
**Sex (Reference: Male)**			
Vaccine effect	-2.16	0.170	<0.0001
Sex effect	-0.430	0.0884	<0.0001
Interaction effect	-0.663	0.233	0.0045
**Ethnicity (Reference: Arab)**			
Vaccine effect	-1.77	0.136	<0.0001
Ethnicity effect	0.319	0.0982	<0.0001
Interaction effect	-0.289	0.263	0.27
**Month of observation (Reference: Oct-Dec 2020)**			
Vaccine effect	-4.45	0.711	<0.0001
Jan-Apr 2021 effect	-1.62	0.0906	<0.0001
May-Jul 2021 effect	-2.40	0.236	<0.0001
Interaction Jan-Apr 2021	2.89	0.722	<0.0001
Interaction May-Jul 2021	3.77	0.809	<0.0001

Outcome: Death

	Coefficient estimate	Standard error	P-values
**Comorbidity (Reference: Without comorbidity)**			
Vaccine effect	-0.262	0.519	0.614
Comorbidity effect	5.40	0.482	<0.0001
Interaction effect	-3.40	0.694	<0.0001
**Age (Reference: age < 60)**			
Vaccine effect	-2.54	0.740	0.0006
Age effect	2.86	0.263	<0.0001
Interaction effect	0.911	0.815	0.2640
**Sex (Reference: Male)**			
Vaccine effect	-2.21	0.469	<0.0001
Sex effect	-0.589	0.225	0.0090
Interaction effect	0.745	0.626	0.2340
**Ethnicity (Reference: Arab)**			
Vaccine effect	-1.94	0.355	<0.0001
Ethnicity effect	-0.301	0.297	0.3110
Interaction effect	0.459	0.722	0.5250
**Month of observation (Reference: Oct-Dec 2020)**			
Vaccine effect	-16.2	0.308	<0.0001
Jan-Apr 2021 effect	-1.37	0.207	<0.0001
May-Jul 2021 effect	-0.943	0.308	0.0022
Interaction Jan-Apr 2021	14.5	0.350	<0.0001
Interaction May-Jul 2021	15.2	0.586	<0.0001

P-value for testing the main and interaction effects between each risk factors and vaccine status. P-values were calculated using two-sided tests with no adjustment for multiple comparisons, and were computed using the Wald test on the coefficients of the Cox proportional hazard model. P-values < 0.05 are considered statistically significant.

Supplementary Table 9. Effectiveness of the Vaccine in Preventing Severe COVID-19 Outcomes stratified according to different risk factors: Sensitivity analysis

Characteristic	Hospitalization			Critical care admission			Death		
	**Effectiveness (%)**	**95% CI**	**p-value***	**Effectiveness (%)[1]**	**95% CI[1]**	**p-value***	**Effectiveness (%)[1]**	**95% CI[1]**	**p-value***
**Month of observation n (%)**			**<0.0001**			**<0.0001**			**<0.0001**
Oct-Dec 2020	97.3	95.7 , 98.3		98.8	95.3 , 99.7		100	100 , 100	
Jan-Apr 2021	73.3	70.6 , 75.7		79.1	73.1 , 83.7		81.9	66.9 , 90.1	
May-Jul 2021	34.6	14.2 , 50.2		49.6	0 , 76.4		62.5	31.4 , 79.5	
**Comorbidity**			**<0.0001**			**0.007**			**<0.0001**
Without any comorbidity	66.9	61.9 , 71.3		74.6	62.2 , 83		23	0 , 72.2	
With one or more comorbidity	81.3	79.2 , 83.2		87	82.8 , 90.2		97.4	92.9 , 99.1	
**Ethnicity**			0.234			0.270			0.525
Arab	76.7	74.3 , 78.9		82.9	77.7 , 86.9		85.6	71.1 , 92.8	
Non-Arab	79.4	75.5 , 82.6		87.2	80.1 , 91.8		77.2	21.8 , 93.4	
**Age group**			**<0.0001**			0.697			0.264
≤ 60 years	82.7	80.6 , 84.5		84.9	79.6 , 88.8		92.1	66.4 , 98.2	
More than 60 years	66.3	61.7 , 70.3		83.4	76.6 , 88.3		80.4	61.8 , 89.9	
**Sex**			**0.0023**			**0.0045**			0.234
Female	79.9	77.4 , 82.1		77.6	69.4 , 83.7		76.8	47.6 , 89.8	
Male	73.8	70.2 , 76.9		88.5	83.9 , 91.7		89	72.4 , 95.6	

CI = Confidence Interval. P-value for testing the interaction between risk factors and vaccine status. Only statistically significant interactions (marked in bold) can be interpreted as significant subgroup differences. P-values were calculated using two-sided tests

with no adjustment for multiple comparisons, and were computed using the Wald test on the interaction term in the Cox proportional hazard model. P-values < 0.05 are considered statistically significant.

Supplementary Table 10. Effectiveness using time varying coefficient model with a step function: Sensitivity analysis

Time period	Hospitalization			Critical care admission		
	**Effectiveness (%)**	**95% CI**	**p-value**	**Effectiveness (%)**	**95% CI**	**p-value**
Prior to day 57	80.5	78.4 , 82.5	<0.001	85.5	80.9 , 89.1	<0.001
After day 57	68.1	63 , 72.4	<0.001	77	65.9 , 84.5	<0.001

HR = Hazard Ratio, CI = Confidence Interval. P-values were calculated using two-sided tests with no adjustment for multiple comparisons, and were computed using the Wald test on the coefficients of the Cox proportional hazard model. P-values < 0.05 are considered statistically significant.

Supplementary Figure 2. Schoenfeld residuals for testing proportionality of the Cox proportional hazard model: Sensitivity analysis

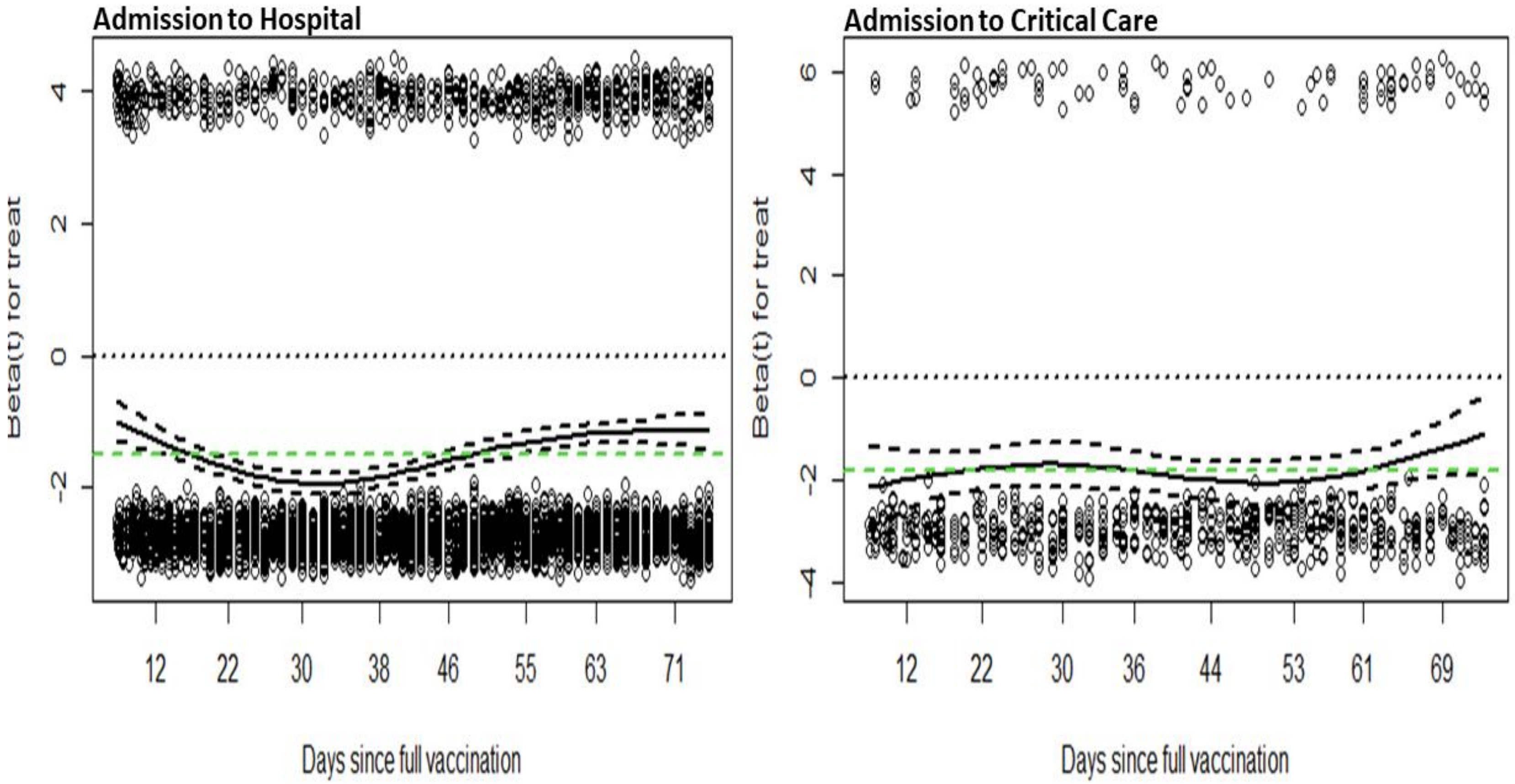

The solid black curve represents the estimated time varying log-hazard ratio with the dashed black curves representing the corresponding 95% confidence limits. The green dashed line represents the estimated log-hazard ratio obtained from the Cox proportional hazard model (under the assumption of proportional hazards).

3 - Ethnicity of the study participants

Supplementary Table 11. Ethnicity of the study participants

**Ethnicity**	**Overall** **n (%)**	**Unvaccinated** **n (%)**	**Vaccinated** **n (%)**
Asian	1,209,118 (55)	605,694 (55)	603,424 (55)
Arab	760,211 (35)	375,887 (34)	384,324 (35)
African	94,290 (4.3)	48,237 (4.4)	46,053 (4.2)
European	61,796 (2.8)	31,090 (2.8)	30,706 (2.8)
Northern_American	25,518 (1.2)	13,461 (1.2)	12,057 (1.1)
Middle_Eastern_Others	16,427 (0.7)	8,485 (0.8)	7,942 (0.7)
Caucasian	11,457 (0.5)	6,203 (0.6)	5,254 (0.5)
Pacific_Islanders_Others	12,050 (0.5)	6,328 (0.6)	5,722 (0.5)
Latin_American	8,905 (0.4)	4,501 (0.4)	4,404 (0.4)
