# Supplementary material for: Effectiveness of BBIBP-CorV vaccine against severe outcomes of COVID-19 in Abu Dhabi, United Arab Emirates
Source: Nat Commun. 2022 Jun 9;13:3215. doi: 10.1038/s41467-022-30835-1 (PMC9184465; doi:10.1038/s41467-022-30835-1)
Supplement: Supplementary file 3 — Reporting Summary [file 41467_2022_30835_MOESM3_ESM.pdf]

nature portfolio

Corresponding author(s): Dr. Nawal Al Kaabi

Last updated by author(s): Apr 1, 2022

Reporting Summary

Nature Portfolio wishes to improve the reproducibility of the work that we publish. This form provides structure for consistency and transparency in reporting. For further information on Nature Portfolio policies, see our Editorial Policies and the Editorial Policy Checklist.

Please do not complete any field with "not applicable" or n/a. Refer to the help text for what text to use if an item is not relevant to your study. For final submission: please carefully check your responses for accuracy; you will not be able to make changes later.

Statistics

For all statistical analyses, confirm that the following items are present in the figure legend, table legend, main text, or Methods section.

n/a	Confirmed	
[ ]	[x]	The exact sample size (*n*) for each experimental group/condition, given as a discrete number and unit of measurement
[ ]	[x]	A statement on whether measurements were taken from distinct samples or whether the same sample was measured repeatedly
[ ]	[x]	The statistical test(s) used AND whether they are one- or two-sided<br>*Only common tests should be described solely by name; describe more complex techniques in the Methods section.*
[ ]	[x]	A description of all covariates tested
[ ]	[x]	A description of any assumptions or corrections, such as tests of normality and adjustment for multiple comparisons
[ ]	[x]	A full description of the statistical parameters including central tendency (e.g. means) or other basic estimates (e.g. regression coefficient) AND variation (e.g. standard deviation) or associated estimates of uncertainty (e.g. confidence intervals)
[ ]	[x]	For null hypothesis testing, the test statistic (e.g. *F*, *t*, *r*) with confidence intervals, effect sizes, degrees of freedom and *P* value noted<br>*Give P values as exact values whenever suitable.*
[x]	[ ]	For Bayesian analysis, information on the choice of priors and Markov chain Monte Carlo settings
[x]	[ ]	For hierarchical and complex designs, identification of the appropriate level for tests and full reporting of outcomes
[x]	[ ]	Estimates of effect sizes (e.g. Cohen's *d*, Pearson's *r*), indicating how they were calculated

Our web collection on statistics for biologists contains articles on many of the points above.

Software and code

Policy information about availability of computer code

Data collection	The data was collected using Cerner millennium – Health Information system, Power Bi version and excel<br>was used to retrieve data from electronic health records. Cerner Millennium Software, Health Information system – Version 3.30 Build #2020-10-23T13:46:35Z<br>Power Bi Desktop , July 2021 Update (2.95.804.0), Microsoft Excel 2016 for Microsoft 365 MSO (16.0.5278.1000) 64-bit
Data analysis	All statistical analyses were performed using R software version 4.0.4 and 4.1.0

For manuscripts utilizing custom algorithms or software that are central to the research but not yet described in published literature, software must be made available to editors and reviewers. We strongly encourage code deposition in a community repository (e.g. GitHub). See the Nature Portfolio guidelines for submitting code & software for further information.

Data

Policy information about availability of data

All manuscripts must include a data availability statement. This statement should provide the following information, where applicable:

- Accession codes, unique identifiers, or web links for publicly available datasets
- A description of any restrictions on data availability
- For clinical datasets or third party data, please ensure that the statement adheres to our policy

Clinical, demographic, and vaccination data of all subjects were extracted from the Abu Dhabi Health Services Company (SEHA) electronic health database and the Abu Dhabi health information exchange platform (Malaffi), which is a centralized electronic database of patient health information.
According to Department of health regulations, individual-level data cannot be shared openly.
Specific requests for remote access to de-identified data should be referred to DOH, Research committee.

Requests sent to IRB, DOH medical research and development division (medical.research@doh.gov.ae) will be considered within 21 days pending IRB approval and DOH regulations.
Auxiliary and summary data generated from the analyses are available in the supplementary file

Field-specific reporting

Please select the one below that is the best fit for your research. If you are not sure, read the appropriate sections before making your selection.

[x] Life sciences [ ] Behavioural & social sciences [ ] Ecological, evolutionary & environmental sciences

Life sciences study design

All studies must disclose on these points even when the disclosure is negative.

Sample size	It is a population-based study in Abu Dhabi in which all participants vaccinated prior to 1 July 2021 and all unvaccinated controls prior to 1 July 2021 were included.<br>This provided us with a sufficient number of participants (over 3 million) to estimate vaccine effectiveness
Data exclusions	The exclusion criteria included individuals less than 18 years of age. They were excluded based on preset exclusion criteria as the study focused on effectiveness in adults. Vaccinated individuals with time interval between 1st and 2nd vaccine dose less than two weeks were excluded from this analysis, along with those who had a history of COVID-19 hospitalization prior to their baseline date. As the time interval between two doses of the vaccine and previous history of COVID-19 affects the effectiveness of the vaccine they were excluded from the analysis
Replication	No experiments were carried out in this study. It was a retrospective study in which the data were already available in the electronic health record database and the variables of interest for this study was retrieved from the records. Data analysis was verified for accuracy. However, all the results of the study can be replicated using the R code which is publicly shared at the following link: https://github.com/abderrahimoulhaj/vaccine_effectiveness_nature_communications.git
Randomization	No randomization was done as it was not a RCT, however each vaccinated individual was matched to an unvaccinated control in 1:1 ratio using the Rolling Entry Matching (REM) according to their age, sex, nationality and comorbidities
Blinding	It is a retrospective cohort study, in which the outcomes of the study (hospitalization, critical care admissions and death) were already documented. The data was only extracted for the study purpose. To further avoid any bias in extraction, the clinical, demographic, and vaccination data of all subjects were extracted from the Abu Dhabi Health Services Company (SEHA) electronic health database separately and the patient health information were extracted from Abu Dhabi health information exchange platform (Malaffi) separately. Only a third person combined both the data based on the identification numbers.

Reporting for specific materials, systems and methods

We require information from authors about some types of materials, experimental systems and methods used in many studies. Here, indicate whether each material, system or method listed is relevant to your study. If you are not sure if a list item applies to your research, read the appropriate section before selecting a response.

Materials & experimental systems

n/a	Involved in the study	
[x]	[ ]	Antibodies
[x]	[ ]	Eukaryotic cell lines
[x]	[ ]	Palaeontology and archaeology
[x]	[ ]	Animals and other organisms
[ ]	[x]	Human research participants
[x]	[ ]	Clinical data
[x]	[ ]	Dual use research of concern

Methods

n/a	Involved in the study	
[x]	[ ]	ChIP-seq
[x]	[ ]	Flow cytometry
[x]	[ ]	MRI-based neuroimaging

Human research participants

Policy information about studies involving human research participants

Population characteristics	Participants were adults who are ≥ 18 years, the median age was 35 years and vaccinated subjects are those who completed their second dose of the vaccine prior to July 1st 2021 and unvaccinated subjects are those who did not receive any dose of the COVID-19 v<br>accination prior July 1st 2021. 2,223,868 individual health records were analyzed of which 64% were male and 36% were female.

Recruitment	Participants were recruited from data on electronic health records using Rolling Entry Matching procedure which matched 1,153,515 vaccinated individuals in a 1:1 ratio to 1,153,515 unvaccinated controls according to age, gender, nationality, comorbidities and the date of entry into the study.
Ethics oversight	This study was approved by the Institutional Review Board of the Department of Health, Abu Dhabi. Approval number: DOH/CVDC/2021/658.

Note that full information on the approval of the study protocol must also be provided in the manuscript.

This checklist template is licensed under a Creative Commons Attribution 4.0 International License, which permits use, sharing, adaptation, distribution and reproduction in any medium or format, as long as you give appropriate credit to the original author(s) and the source, provide a link to the Creative Commons license, and indicate if changes were made. The images or other third party material in this article are included in the article's Creative Commons license, unless indicated otherwise in a credit line to the material. If material is not included in the article's Creative Commons license and your intended use is not permitted by statutory regulation or exceeds the permitted use, you will need to obtain permission directly from the copyright holder. To view a copy of this license, visit http://creativecommons.org/licenses/by/4.0/
